# The efficacy of transcranial magnetic stimulation on migraine: a meta-analysis of randomized controlled trails

**DOI:** 10.1186/s10194-017-0792-4

**Published:** 2017-08-22

**Authors:** Lihuan Lan, Xiaoni Zhang, Xiangpen Li, Xiaoming Rong, Ying Peng

**Affiliations:** 1 Sun Yat-Sen University, Guangzhou, 510288 China; 2 Department of Neurology, Sun Yat-Sen Memorial Hospital, Sun Yat-Sen University, Number 33, Yingfeng Road, Haizhu District, Guangzhou, 510288 China

**Keywords:** Migraine, Transcranial magnetic stimulation, Randomized control trail

## Abstract

**Objectives:**

As a non-invasive therapy, whether transcranial magnetic stimulation (TMS) is effective on migraine. This article was aimed to assess the efficacy of TMS on migraine based on randomized controlled trails (RCTs).

**Methods:**

We searched PubMed, Embase and Cochrane Library electronic databases for published studies which compared TMS group with sham group, conducted a meta-analysis of all RCTs.

**Results:**

Five studies, consisting of 313 migraine patients, were identified. Single-pulse transcranial magnetic stimulation is effective for the acute treatment of migraine with aura after the first attack (*p* = 0.02). And, the efficacy of TMS on chronic migraine was not significant (OR 2.93; 95% CI 0.71–12.15; *p* = 0.14).

**Conclusions:**

TMS is effective for migraine based on the studies included in the article.

## Background

Recently, the incidence of migraine is gradually rising and becoming one of the most common nervous system diseases in the world [1]. According to ICHD-3 (beta version), migraine is divided into migraine without aura, migraine with aura, chronic migraine, complications of migraine, probable migraine and episodic syndromes that may be associated with migraine. Particularly, chronic migraine cause serious damage in the quality of life. However, the effect of the drug therapies, include acute therapies (non-steroidal anti-inflammatory drugs, ergotamine preparations and triptans) and preventive therapies (β-blockers, anticonvulsants, tricyclic antidepressants and calcium channel modulators), are not significantly improved the clinical symptoms.

Transcranial magnetic stimulation (TMS), which is a magnetic field created by an electrical current through a coil wrapped around the scalp or skull. The types of TMS include single-pulse TMS, pair-pulses TMS and repetitive TMS. In neurophysiology, TMS can measure neural conduction, facilitate or inhibit the electrical activity of cerebral cortex [2]. TMS is a noninvasive technology and the first transcranial magnetic stimulator was introduced to the world in 1984–1985 [3]. Nevertheless, using TMS for a therapy was firstly reported on drug-resistant depressed patients in 1996 [4]. After 30 years later, TMS now can be applied for a diagnostic therapy in many diseases including multiple sclerosis, movement disorder, stroke, epilepsy and so on [5, 6]. Meanwhile, TMS also can be used for a therapy. There has been reported a series of diseases covering psychiatric disorders (depression, acute mania, schizophrenia, bipolar disease, panic disorder, post-traumatic stress disorder, substance abuse) and neurologic disorders (Parkinson’s disease, dystonia, tinnitus, epilepsy, stroke) improved by TMS [7–26]. Furthermore, single-pulse and paired-pulse/double-coil TMS are safety for normal human subjects and patients who suffer from migraine [2, 27]. However, there is less randomized control trails (RCTs) to identify the efficacy of TMS in migraine at present. Recently, there are some papers reviewed the effect of TMS for migraine [28, 29], but lack a meta-analysis. Although there is a meta-analysis about noninvasive brain stimulation in migraine, it reached a conclusion that TMS did not reveal significant effects for any outcome [30], moreover, some new RCTs have revealed that TMS is efficacy for migraine recently.

For the exact mechanism of migraine does not exist so far. It may relate to neural and vascular causes, involving cerebral cell hyper excitability, sensitization of the trigeminovascular pathway, correlative predisposing genes and environmental factors. As for migraine with aura, cortical spreading depression (CSD) proved to be its pathogenesis [31–33]. CSD, an inhibition zone of cortical activity after stimulating vertebrate’s cerebral cortex, and the zone would move to adjacent cortex at a speed of 2-5 mm/min. CSD may change the cerebral blood flow and result in headache. Currently, there are evidence that single pulse-TMS can suppress CSD in animal experiment [34]. Correspondingly, some clinical trials are developed to verify whether TMS is effective for migraine. This article provides an update on the effect of TMS in migraine from randomized control trails.

## Methods

According to the Preferred Reporting Items for Systematic Reviews [35], a protocol of study-search strategies, outcome measurements, and methods of statistical analysis was prepared in advance.

### Study-search strategy

In April 2017, the PubMed, Embase and the Cochrane Library electronic databases were researched in the following medical subject headings (MeSH): [Title/Abstract] “Migraine Disorders”, “Migraine”, “Migrain*”, “Transcranial Magnetic Stimulation” and “Randomized”, “Randomized Study”. Moreover, the related articles function was also used to broaden the search, and all studies, abstracts were reviewed without restriction to regions, publication types, or languages.

### Selection criteria

1. The study was a randomized controlled trail that compared transcranial magnetic stimulation with sham group; 2. The study had the quantitative outcomes.

### Data extraction

The data was extracted by two independent authors (Lihuan, L and Xiaoni, Z) as following: (1) Study design. (2) Number of patients in active group and sham group. (3) Device and treatment parameters. (4) The change of headache frequency.

Furthermore, all controversies were settled by consensus. In regard to incomplete data, we contacted the author for detail information. While, there is one study missing the original data (Chiara Rapinesi et al.) [36].

### Quantitative and statistical analysis

For the level of evidence, the Cochrane risk of bias tool was used to evaluate the quality of RCTs [37]. And the odds ratio (OR) was used to assess dichotomous variables, with results being reported using 95% confidence intervals (CI). Heterogeneity within study results was evaluated using Chi squared test and *I* ^*2*^ statistic. Higher χ^2^ and I^2^ statistic manifests more heterogeneity among studies. If *p* value was more than 0.1 and I^2^ was less than 50%, a random-effect model was used. If not, a fixed-effect model was used [37].

Risk of bias summary was used to evaluate if there were potential publication bias. All statistical analyses were done by Review Manager 5.0 (Cochrane Collaboration).

## Results

**Fig. 1:**
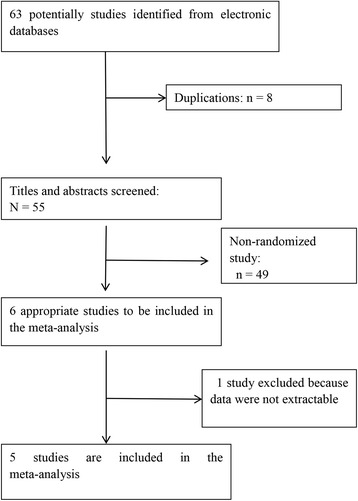
Flow diagram of search strategy

The characteristics of included studies are described in Table 1. Sixty three studies were screened using predefined search strategy (Fig. 1). Eight were excluded as duplication. Forty nine were excluded because of non-randomized study. And 1 was excluded for lacking of exact data. Five studies, consisting of 313 migraine patients, contributed to this meta-analysis [38–42]. And the earliest was reported in 2004, the latest study was published in 2016.

**Table 1:** Characteristics of included studies

Study	Design	Patients		Device	Dose and frequency	Inclusion criteria	Exclusion criteria
		TMS	Sham				
Richard et al. [39]	Randomized, double-blind, sham-controlled	82	82	Portable sTMS over occiput	2 pulses about 30s	MA: diagnosis according to ICHD(2 edition)	MA: aura > 60 min; metal implants; headache due to trauma or over use of drug.
Hatem et al. [40]	Randomized, open-label	12	14	Tabletop clinic- based rTMS over the left motor cortex	12 rTMS sessions at 10 Hz	CM: diagnosis according to ICHD -third edition-III (beta version).	Headache due to over use of drug; other chronic primary/secondary headaches. Use of headache prophylaxis medication within 4 weeks of baseline, comorbid, psychiatric disorders, symptomatic headache, “demonstrable structural lesion by brain magnetic resonance imaging”
Adriana et al. [41]	Randomized, double-blind, parallel-group	7	7	Tabletop clinic- based rTMS-DLPFC	23 sessions of active rTMS-DLPFC, total of 1600 pulses per session	18–80 years; CM: diagnosis according to ICHD (2 edition).	Inability to comply. Other neurologic disorder. Contraindications to TMS. Psychotic symptom or bipolar disorder. Drug or alcohol dependent. Pregnancy. Use of drug that interfere on CE. Severe major depression. Changes in prophylactic medications.
Usha et al. [42]	Randomized, placebo-controlled	47	48	Tabletop clinic- based rTMS over left frontal cortex	10 Hz rTMS, 600 pulses in 10 trains	MP above the age of 15 years has >4 attacks/month in the last 3 months.	Liver or kidney failure, malignancy, uncontrolled hypertension, seizure, structural brain lesion, focal neurological deficit, metal implant, pregnancy.
Filippo et al. [38]	Randomized, double-blind, controlled	6	5	Tabletop clinic- based rTMS-DLPFC	12 rTMS sessions, each rTMS session consisted of 10 trains of 2-s duration, separated by 30-s pause, given at 20-Hz frequency and 90% MT intensity	CM: diagnosis according to IHS [45].	Hamilton scale ≥ 7

*TMS* transcranial magnetic stimulation, *sTMS* single-pulse transcranial magnetic stimulation, *rTMS* repetitive transcranial magnetic stimulation, *MA* migraine with aura, *ICHD* International Classification of Headache Disorder, *CE* cortical excitability, *DLPFC* dorsolateral prefrontal cortex, *MT* motor threshold, *HIS* International Headache Society

### Sensitivity analysis and publication bias

**Fig. 2:**
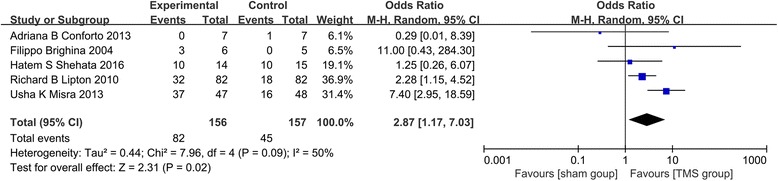
Heterogeneity among studies and the effect of TMS on migraine

**Fig. 3:**
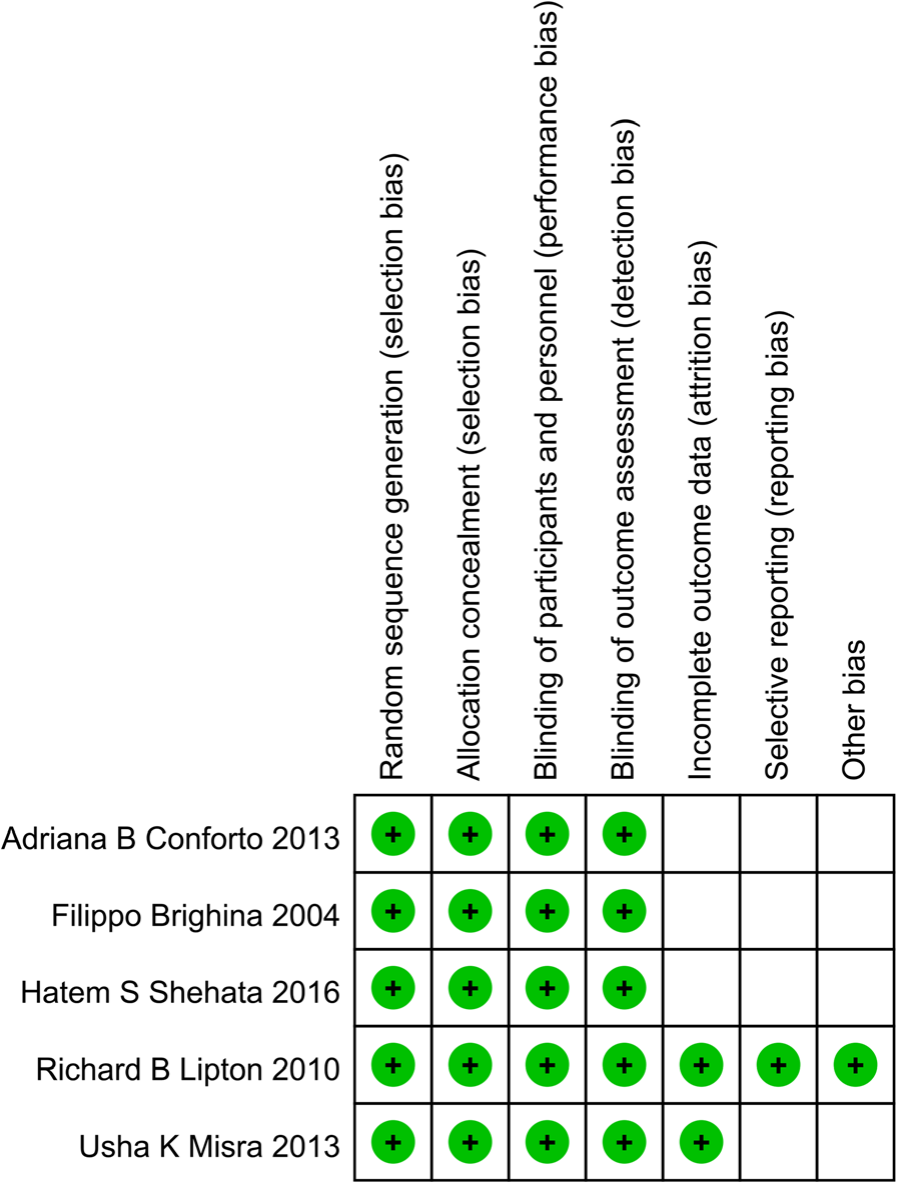
Risk of bias summary of researches included in this meta-analysis

All studies are RCTs, the result of heterogeneity among studies are showed in Fig. 2. Four studies were related to chronic migraine, one study researched TMS for acute treatment of migraine with aura. For all studies, the heterogeneity is as following: χ^2^ = 7.96, *P* = 0 .09, I^2^ = 50%. While, when we combined 4 chronic migraine studies, excepted migraine with aura, the heterogeneity change to: χ^2^ = 6.49, *P* = 0 .09, I^2^ = 54%. Given the small sample sizes in this study, the change of heterogeneity is slight significance. Figure 3 shows risk of bias summary of researches included in this meta-analysis.

### Effect on migraine with aura **(**Fig. 4a**)**

There was one RCT (Richard B Lipton et al.) assessed the efficacy of TMS on migraine with aura. According to the study, more patients were pain-free at 2 h post-treatment and there is significant that single-pulse transcranial magnetic stimulation is effective for the acute treatment of migraine with aura after the first attack (*p* = 0.02).

**Fig. 4:**
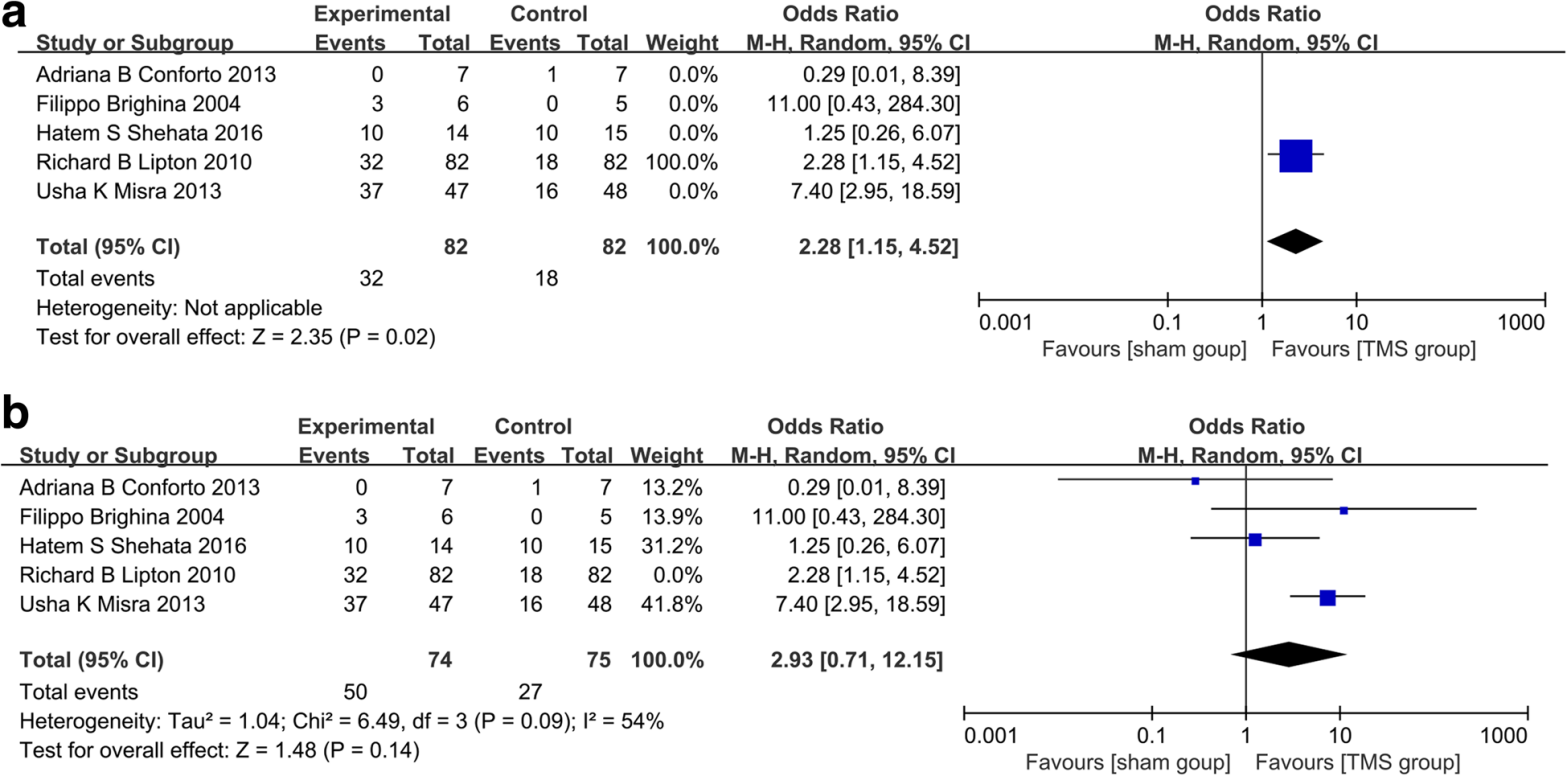
**a** The effect of TMS on migraine with aura. **b** The effect of TMS on chronic migraine

### Effect on chronic migraine **(**Fig. 4b**)**

There were 4 RCTs researched the effect of TMS on chronic migraine. And statistical heterogeneity was detected among the trails (χ^2^ = 6.49, *p* = 0 .09, I^2^ = 54%). Moreover, the efficacy of TMS on chronic migraine was not significant (OR 2.93; 95% CI 0.71 – 12.15; *p* = 0.14).

### Effect on migraine **(**Fig. 2**)**

For all studies, significant statistical heterogeneity was detected (χ^2^ = 7.96, *p* = 0 .09, I^2^ = 50%). And statistically significant effect of group (TMS group, control group) was found by analyzing all trials (OR 2.87; 95% CI 1.17 – 7.03; *p* = 0.02).

## Discussion

Migraine is a kind of chronic headache relating to cortical excitability. As a noninvasive therapy, TMS can activate (or suppress) the cortex excitability. In 2013, a Statement from the European Headache Federation indicates that using of a noninvasive therapy in chronic headaches is not evidence based at present and a neurostimulator should be considered only all alternative drug and behavioural therapies as recommended by international guidelines have failed and medication overuse headache is excluded, due to the lack of proper RCTs [43]. Because there are limited drugs that can improve the quality of life for people with the migraine, and TMS as a promising therapy which can facilitate or inhibit the electrical activity of cerebral cortex and there are some existing RCTs reveal that TMS can relieved headache. Nonetheless, there are few meta-analyses about the effect of TMS for migraine. By combining RCTs and meta-analysis, we hope to evaluate whether TMS can relieve headache and to expand its clinical application. So we decide to assess the effect of TMS on migraine by synthesizing evidences.

In this meta-analysis, 5 RCTs including 313 patients comparing the efficacy of TMS group with control group indicated that TMS was significantly effective for migraine. However, the doses and frequency of TMS in these RCTs were different. And which doses could help to improve the headache frequency of migraine doesn’t reach common understanding. In an open labeled study, Usha et al. reported that high frequency repetitive transcranial magnetic stimulation (rTMS) was effective and well tolerated for migraine prophylaxis [42]. Besides, in other study, M Teepker et al. reported that no statistically significant difference between low-frequency rTMS with sham stimulation was found [44]. In the present meta-analysis, 5 RCTs used a higher frequency (≥ 10 Hz) stimulation. So we considered that higher frequency stimulation may reach an obvious effect. However, the reasons for variability are not only the dose but also the side, location of stimulation, type of coil and the number of sessions. The 5 RCTs were generally delivered at different frequency with a figure-eight coil positioned over the left motor cortex. Due to the difference in the side, location of stimulation, type of coil and the number of sessions, the efficacy of magnetic signal on electrical activity of cerebral cortex is different. Nevertheless, there is not a common standard of TMS on migraine at present. Given that, future well-designed RCTs are needed to confirm which dose, side, and location of stimulation, type of coil or the number of sessions is more effective for migraine.

Transcranial magnetic stimulation, a novel treatment method, is considered to be effective for migraine in this meta-analysis. However, there is an inevitable problem that these RCTs did not have a standard control group. In four RCTs, there were active TMS group and sham TMS group [38, 39, 41, 42]. In one RCT, there were active TMS group and botulinum toxin-A injection group [40]. Therefore, we have no idea that whether TMS is superior to conventional therapy. It is necessary to conduct more clinical trials to assess the efficacy of TMS on migraine in the future.

When evaluated the effect of TMS on chronic migraine, we reached a conclusion that there was not statistically significant difference in effect between active TMS group and sham TMS group. In light of this, we put forward two hypotheses: firstly, chronic migraine is a chronic pathogenic process and the threshold of pain had been raised. Although TMS can change the excitability of cortex, it needs more time to do this. Secondly, due to the small sample, this conclusion was not definite. Future well-designed RCTs are needed to confirm this conclusion.

Besides, this meta-analysis has some limitations as following: first, the main limitation is that we only included published data and there were 5 RCTs included in this article. The published bias comes to an unavoidable issue. Therefore, the conclusion came from synthesizing evidence should be considered with caution. And in order to improve the reliability of this meta-analysis, we only take RCT into account. Although a meta-analysis of RCTs can provide a more reliable result, due to the lack of studies, only 5 RCTs included in this meta-analysis prevented us from reaching a more authentic outcome. Second, for all studies included in the analysis, patients were not a grouped by severity of pathogenic condition, sexuality or age and so on. So the efficacy of TMS should be taken into consideration. Third, the patients included in this paper mostly came from general hospitals or major institutions, so the patients might not represent patient populations in the world. Fourth, due to the difference of original data on studies included in the analysis, this meta-analysis did not make full use of data in studies.

## Conclusion

In summary, this meta-analysis indicates that TMS is effective for migraine based on the studies included in the article. For the stimulation parameters, using figure-of-8-shaped coil over the left motor cortex with higher frequency may be effect based on the studies included in the article. However, because of above limitations, the efficacy of TMS on migraine should be tasted on more RCTs in the future.

## Abbreviations

CI: Confidence intervals
CSD: Cortical spreading depression
MeSH: Medical subject headings
OR: Odds ratio
RCTs: Randomized controlled trails
rTMS: repetitive transcranial magnetic stimulation
TMS: Transcranial magnetic stimulation
